# Interpretable machine learning model based on routine metabolic laboratory indices to identify advanced chronic kidney disease

**DOI:** 10.3389/fendo.2026.1776419

**Published:** 2026-03-18

**Authors:** Baoye Ye, Xikui Zhang, Weikun Zhu, Zhongfu Xiao, Shuhui Huang

**Affiliations:** 1 The Second Affiliated Hospital of Fujian University of Traditional Chinese Medicine, Fuzhou, China; 2 Fujian University of Traditional Chinese Medicine, Fuzhou, China

**Keywords:** chronic kidney disease, machine learning, metabolic dysregulation, model interpretability, routine laboratory biomarkers

## Abstract

**Introduction:**

Early identification of advanced chronic kidney disease (CKD), a condition accompanied by profound metabolic and endocrine disturbances, is essential for timely nephrology referral and intervention. However, widely used risk equations often require albuminuria or repeated measurements that are not consistently available in routine clinical practice.

**Methods:**

We retrospectively analyzed adult patients from three different departments affiliated to one university, including two independent hospitals and a clinic department. Routinely collected demographic, clinical, and metabolic laboratory variables were used to develop machine learning models for distinguishing preserved kidney function (CKD G1–2) from advanced stages (G3a–5). Five algorithms were trained and internally validated in a development cohort, followed by external validation in an independent cohort. Model performance was assessed by discrimination, calibration, and interpretability using feature importance and SHAP (Shapley Additive Explanations).

**Results:**

Among 308 patients in the development cohort and 52 in the external cohort, the Gradient Boosting classifier achieved the best discrimination (AUC = 0.972 internally; 0.965 externally) with good calibration. Urea, kidney disease type, phosphorus, albumin, and lipid-related parameters–reflecting systemic metabolic dysregulation–emerged as key contributors to model predictions.

**Discussion:**

An interpretable Gradient Boosting model leveraging routinely measured metabolic laboratory data accurately identifies advanced CKD and captures clinically meaningful metabolic patterns associated with disease severity, supporting its potential integration into electronic health records for risk stratification and identification of advanced CKD among patients with established CKD in specialist care.

## 1 Introduction

Chronic kidney disease (CKD) affects approximately 10–15% of adults worldwide and represents not only a progressive loss of renal function, but also a systemic disorder characterized by profound metabolic and endocrine disturbances. (1–4). Patients who have progressed to advanced CKD (stages G3a-G5) face particularly high risks and often require timely nephrology referral, optimized renin-angiotensin-aldosterone system blockade, preparation for kidney replacement therapy, and close monitoring (4, 5). With the progression of chronic renal failure, a series of disorders gradually emerge, including hormone production deficiency (e.g., EPO, calcitriol), hormone clearance impairment (e.g., insulin, glucagon, PTH, prolactin), hormone resistance (e.g., insulin, GH), feedback regulation axis dysfunction (e.g., calcium-phosphorus axis, gonadal axis), protein metabolism disorders, lipid metabolism disorders, and acid-base imbalance. These factors collectively lead to a range of classic complications, such as anemia, bone diseases, malnutrition, cardiovascular diseases, sexual dysfunction, and growth retardation.However, CKD is frequently asymptomatic during its early and moderate stages, and many patients are only recognized after significant kidney function decline, limiting opportunities for intervention.

Standard CKD assessment relies on estimated glomerular filtration rate (eGFR) and albuminuria, as recommended by international clinical practice guidelines (6, 7). Several validated equations-most notably the Kidney Failure Risk Equation (KFRE)-accurately predict long-term kidney failure risk across diverse populations (8–10). However, these approaches primarily focus on renal outcomes and often underutilize routinely measured metabolic biomarkers that may capture systemic disease severity. Nevertheless, their applicability in routine practice is limited, because quantitative albuminuria testing and longitudinal follow-up measurements are often unavailable in primary care settings and resource-limited environments (11, 12). Moreover, most existing tools focus on forecasting future kidney failure rather than identifying patients who have already reached advanced CKD and require prioritization for specialist evaluation. A simple, data-efficient tool that relies only on routinely collected laboratory markers to flag individuals with advanced CKD would therefore address a critical unmet need in real-world clinical workflows.

Machine learning (ML) methods offer a powerful framework for integrating multiple clinical and biochemical features and capturing nonlinear relationships that traditional regression models may overlook (13). Such methods are particularly well suited for integrating multidimensional metabolic data and uncovering nonlinear patterns related to endocrine–metabolic alterations in CKD. In nephrology, ML has been increasingly used for predicting incident CKD, rapid eGFR decline, dialysis initiation, and mortality, often achieving improved discrimination compared with conventional models (14, 15). However, many published ML-based studies rely on high-dimensional electronic health record (EHR) data, specialized biomarkers, or imaging modalities, which limit their feasibility in routine clinical settings (16, 17). External validation is also lacking in a substantial proportion of these studies, and concerns about model interpretability further hinder widespread adoption (18).

In contrast to conventional CKD assessment based primarily on serum creatinine and eGFR, advanced CKD is accompanied by profound systemic metabolic and endocrine disturbances involving protein metabolism, lipid remodeling, nutritional status, and mineral balance. These alterations may provide complementary information on disease severity that is not fully captured by renal filtration markers alone.

Therefore, the aim of the present study was not to replace eGFR-based staging, but to develop and externally validate an interpretable machine learning model that leverages routinely measured metabolic laboratory indices to complement conventional assessment. By integrating multidimensional biochemical features and modeling their nonlinear interactions, this approach seeks to identify patients with advanced CKD within specialist nephrology care settings where longitudinal eGFR data or albuminuria measurements may be unavailable or incomplete. This work is intended as a diagnostic classification tool at the point of testing and is not designed to provide longitudinal prognostic predictions of CKD progression. Importantly, the present study was conducted exclusively in adult patients with established CKD receiving specialist care, and the proposed model is not intended for general population screening.

## 2 Materials and methods

### 2.1 Study design and population

This retrospective diagnostic modeling study included adult patients with chronic kidney disease (CKD) from three independent clinical institutions. All enrolled patients met the diagnostic criteria for chronic kidney disease(≥3 months of abnormal kidney function per KDIGO).The development cohort comprised CKD patients consecutively recruited from a tertiary teaching hospital (the Second Affiliated Hospital of Fujian University of Traditional Chinese Medicine) between 2020.1 and 2025.1 and was used for model training and internal validation.Most patients in the model are taking regular treatment to treat their basic diseases including hypertenson-controling, blood sugar-controling, lipid-lowering, phosphate-binding therapy, et al. An independent external validation cohort consisted of consecutive patients from another tertiary teaching hospital (the Third Affiliated Hospital of Fujian University of Traditional Chinese Medicine) and an outpatient clinic (Guo Yi Tang Fujian University of Traditional Chinese Medicine) during 2025.1–2025.10. This design enabled evaluation of the model’s generalizability across different clinical settings and time periods. All three institutions used routine clinical laboratory tests in daily practice. To ensure data comparability across centers, key laboratory measurements were harmonized by unifying measurement units and confirming consistency of assay methods where applicable. This quality control process minimized potential inter-institutional variability in laboratory data.

The eligibility criteria were aligned with contemporary CKD guideline-based definitions that prioritize reliable serum creatinine and routine biochemical testing (6, 19). Adults aged ≥18 years with same-day measurements of serum creatinine and standard laboratory panels—including lipid profiles, protein-related markers, electrolytes, and urinalysis—were included. Patients with missing key predictor variables such as serum creatinine, age, or sex were excluded, consistent with established best practices for diagnostic model development in nephrology (20).

The study protocol was approved by the ethics committees of the participating institutions, and the requirement for written informed consent was waived due to the retrospective design and use of anonymized data.

### 2.2 Outcome definition

Serum creatinine measurements were performed in each center using standardized enzymatic assays traceable to isotope-dilution mass spectrometry, ensuring calibration consistency across laboratories. For analysis, creatinine values reported in μmol/L were converted to mg/dL by dividing by 88.4.

1Estimated glomerular filtration rate (eGFR) was calculated using the 2009 CKD-EPI creatinine equation, which remains one of the most widely validated formulas for kidney function estimation in diverse populations (21, 22). Sex-specific and age-adjusted coefficients were applied without race adjustments, consistent with recent consensus recommendations (19) (see Equation 1). The equation is shown below:

(1) $$\text{eGFR}=141\times \text{min}{\left(\frac{\text{Scr}}{\kappa },1\right)}^{\alpha }\times \text{max}{\left(\frac{\text{Scr}}{\kappa },1\right)}^{-1.209}\times 0.993^{\text{Age}}\times \text{SexFactor}$$

CKD stage was defined according to eGFR categories: G1 (eGFR ≥90 mL/min/1.73 m^2^), G2 (60–89 mL/min/1.73 m^2^), G3a (45–59 mL/min/1.73 m^2^), G3b (30–44 mL/min/1.73 m^2^), G4 (15–29 mL/min/1.73 m^2^) and G5 (<15 mL/min/1.73 m^2^). For the purpose of model development, we dichotomised CKD stages into preserved kidney function (G1-G2) versus advanced CKD (G3a-G5). A binary outcome variable (CKD_binary) was therefore created, with 0 indicating G1-G2 and 1 indicating G3a-G5. This binary label served as the target variable for all machine learning classifiers (Equation 2). The binary outcome was defined as:

(2) $$\text{CKD binary}=\left\{ \begin{array}{l} \begin{array}{cc} 0, & \text{if CKD stage}\in \left\{\text{G}1,\text{G}2\right\}, \end{array}\\ \begin{array}{cc} 1, & \text{if CKD stage}\in \left\{\text{G}3\text{a},\text{G}3\text{b},\text{G}4,\text{G}5\right\}. \end{array} \end{array}\right.$$

Accordingly, the modeling objective was cross-sectional classification of advanced CKD status at the index encounter, rather than prediction of future disease progression.

### 2.3 Candidate predictors and data preprocessing

We considered routinely available clinical and laboratory variables as candidate predictors, including blood pressure history, lipid markers, protein-related indices, electrolytes, uric acid, and dipstick urinalysis. These variables align with established CKD risk factors reported in large epidemiological and mechanistic studies (23, 24). The inclusion of lipid profiles, albumin, uric acid, calcium, and phosphorus is supported by evidence demonstrating their association with CKD severity, inflammation, mineral–bone disorder, and progression risk (25). Importantly, serum creatinine, age, and sex were used exclusively for eGFR calculation and outcome definition (advanced CKD vs. preserved kidney function) and were intentionally excluded from the model input feature set to reduce the risk of outcome circularity.

Duration of kidney disease and all biochemical indices included in the model were treated as continuous variables and converted to numeric type in Python, coercing non-numeric entries to missing. Missing values in continuous variables were imputed using within-cohort medians, consistent with best-practice guidelines for clinical prediction modeling (26). Categorical predictors such as primary kidney disease type, hypertension, diabetes, proteinuria, hematuria, and residence were treated as factors.Primary kidney disease type and residence were included to capture etiological and contextual heterogeneity within the study population. We acknowledge that these variables may not be uniformly defined or routinely collected across healthcare systems; therefore, they should be regarded as context-dependent and optional predictors rather than universally required inputs. Notably, interpretability analyses indicated that the dominant predictive signal was driven by routinely measured laboratory variables. Missing categorical values were imputed as an explicit “Missing” category to avoid discarding samples. This approach was selected *a priori* as a pragmatic and reproducible strategy to preserve sample size and to limit instability in model training given the moderate cohort size. Median imputation is robust to outliers and does not assume a specific distribution, while the explicit “Missing” category allows categorical missingness to be carried forward without discarding patients. A formal sensitivity analysis comparing alternative imputation methods was not conducted in the current study and is therefore acknowledged as a limitation (see Section 4.4).

To enable downstream ML algorithms requiring numerical inputs, categorical variables were one-hot encoded using the development cohort as reference. Dummy variables were generated for each category except the first level to avoid collinearity. The same encoding scheme was subsequently applied to the internal test subset and external validation cohort by aligning columns and filling absent categories with zeros. This ensured that all feature matrices retained an identical structure, consistent with recommended standards for reproducible ML pipelines (27). No additional scaling was applied since the laboratory features were already on comparable clinical scales. Finally, the development cohort was randomly split into a 70% training set and a 30% internal test set using stratification by the CKD_binary label to preserve outcome distribution. This single stratified split was used to ensure independence between training and internal test subsets and to maintain identical data partitions across all candidate models for fair algorithm comparison.

### 2.4 Machine learning model development

Five supervised machine learning algorithms were developed using the processed feature matrices: Random Forest (RF), Gradient Boosting (GB), Extra Trees (ET), Logistic Regression (LR), and an Artificial Neural Network (ANN). These models represent widely used ensemble-based and parametric approaches that have shown strong performance in structured clinical data prediction tasks (28–31).

The RF classifier was configured with 500 trees, bootstrap aggregation, and class_weight set to “balanced_subsample”. The ET classifier used 500 extremely randomized trees with class_weight set to “balanced”. The GB classifier employed default hyperparameters, including a learning rate of 0.1, following established gradient-boosting implementations in clinical ML studies (32). Gradient Boosting was implemented using default (or near-default) scikit-learn settings. Systematic hyperparameter optimization was not performed in order to maintain a standardized and reproducible comparison across algorithms and to reduce overfitting risk given the moderate sample size; nevertheless, future studies with larger cohorts may explore tuning of learning rate, number of estimators, and tree depth to further improve performance. Logistic regression with L2 regularization using the lbfgs solver (maximum 2,000 iterations) served as the linear baseline model.

The ANN was implemented as a multilayer perceptron with two hidden layers (64 and 32 neurons) and ReLU activation, trained using the Adam optimizer for up to 1,000 iterations—an architecture commonly used for structured healthcare data (33). All models were developed in Python using the scikit-learn library, which provides standardized implementations for reproducible biomedical ML research (34). Model fitting was performed exclusively on the training subset, with stratification ensuring consistent prevalence of advanced CKD across partitions.

We did not perform automated hyperparameter optimization (e.g., grid search, randomized search, or cross-validated tuning). Instead, we adopted a prespecified and reproducible configuration to enable fair comparison across algorithms under standardized conditions. For each model, a small number of key hyperparameters were set to commonly used values based on prior literature and clinical machine learning practice, while remaining parameters followed scikit-learn default settings. All models were trained and evaluated using the same feature set, preprocessing pipeline, stratified 70/30 train–test split, and evaluation metrics. This design was chosen to reduce overfitting risk given the moderate sample size and to ensure that performance differences primarily reflected algorithmic characteristics rather than tuning intensity.

Given the moderate sample size relative to the number of predictors, including one-hot encoded categorical variables, we adopted conservative modeling choices to mitigate overfitting risk. Model complexity was intentionally constrained, and no high-dimensional feature expansion or deep neural architectures were used. External validation in an independent cohort further served as a safeguard against overly optimistic internal performance estimates.

### 2.5 Model evaluation and validation

Model performance was initially assessed using the internal test subset and subsequently evaluated in the external validation cohort. Discriminative ability was quantified using the area under the receiver operating characteristic curve (AUC), a widely accepted metric for diagnostic model assessment in nephrology and machine learning studies (35, 36). Bootstrapped confidence intervals were computed to reduce sampling variability and improve robustness of performance estimation (37).

Using a fixed classification threshold of 0.5, accuracy, precision, recall, F1-score, and Cohen’s kappa were calculated as a reference for standardized comparison across models. In addition, to evaluate the robustness of classification performance across different decision thresholds, a threshold-sensitivity analysis was performed for the Gradient Boosting model in the external validation cohort, examining model performance across a range of probability thresholds (38, 39) (see Equations 3–8). These performance metrics were defined as follows:

(3) $$Accuracy=\frac{TP+TN}{TP+TN+FP+FN}$$

(4) $$Precision=\frac{TP}{TP+FP}$$

(5) $$Recall=\frac{TP}{TP+TN+FP+FN}$$

(6) $$Accuracy=\frac{TP+TN}{TP+FN}$$

(7) $$F1-score=\frac{2\times Precision\times Recall}{Precision+Recall}\;$$

(8) $$Kappa=\frac{P_{p}+P_{exp}}{1-P_{exp}}$$

Cohen’s kappa statistic is widely used to evaluate agreement beyond chance in clinical prediction research (40).

Calibration of the Gradient Boosting classifier—the best-performing model—was assessed using quantile-based deciles to compare mean predicted versus observed event rates, a method aligned with established guidelines for evaluating probability estimates in clinical prognostic modeling (41, 42).

In addition to visual calibration curves, quantitative calibration metrics were computed, including the Brier score and calibration slope. The Brier score summarizes overall probabilistic accuracy as the mean squared difference between predicted probabilities and observed outcomes, while calibration slope was estimated by regressing observed outcomes on the predicted log-odds to quantify agreement between predicted and observed risks. To reduce optimistic bias, these quantitative calibration metrics were calculated and reported for the external validation cohort.

For descriptive comparisons, baseline demographic and biochemical characteristics were examined using Student’s t-test or Mann-Whitney U test for continuous variables, and chi-square or Fisher’s exact test for categorical variables, following standard statistical conventions in nephrology research (43). A two-sided P value<0.05 was considered statistically significant.

### 2.6 Model interpretability and statistical analysis

Interpretability analyses focused on the Gradient Boosting classifier, which demonstrated superior performance across both internal and external evaluations. Global feature importance was computed using impurity-based measures, consistent with widely implemented frameworks for interpreting tree-based ensemble models in biomedical applications (44).

To reduce the risk of outcome circularity arising from variables used in eGFR construction, serum creatinine, age, and sex were intentionally excluded from the model input features from the outset. Interpretability analyses (global feature importance and SHAP) were therefore performed on the Gradient Boosting classifier trained using routinely measured metabolic and clinical variables not directly involved in eGFR calculation. Grouped importance measures were then computed for multi-level categorical variables such as primary kidney disease type and place of residence, following established methods for aggregated predictor evaluation (45).

To further elucidate model behavior, SHapley Additive exPlanations (SHAP) were used to characterize both global patterns and individualized prediction pathways. SHAP is a unified, theoretically grounded framework for interpreting ML models and has been increasingly adopted in clinical prediction research due to its ability to provide consistent, local, and directionally informative attributions (46, 47). SHAP summary plots quantified the magnitude and directionality of each predictor’s contribution across the cohort, while SHAP waterfall plots highlighted the cumulative influence of multiple variables on individual predictions. All visualizations were generated using the SHAP library in Python.

## 3 Results

### 3.1 Patient characteristics

Baseline demographic and clinical characteristics of the development cohort (the Second Affiliated Hospital of Fujian University of Traditional Chinese Medicine) and the external validation cohort (the Third Affiliated Hospital of Fujian University of Traditional Chinese Medicine and Guo Yi Tang Fujian University of Traditional Chinese Medicine) are presented in **Table 1**, and comparisons between CKD G1–2 and G3a-5 within the development cohort are summarized in **Table 2**. The two cohorts demonstrated comparable distributions of age, sex, and major comorbidities, consistent with the stable epidemiological patterns reported in CKD populations (1, 24).

**Table 1:** Baseline demographic and clinical characteristics of patients in the development and external validation cohorts.

Variable	Level	Development cohort	Validation cohort	p-value
A/G		1.5 ± 0.3	1.5 ± 0.3	0.764
ALB		39.7 ± 6.8	41.7 ± 6.3	0.082
ApoA1		1.4 ± 0.4	1.4 ± 0.2	0.931
ApoB		1.1 ± 0.4	1.1 ± 0.4	0.643
CREA		242.9 ± 206.7	232.8 ± 190.2	0.738
Ca		2.2 ± 0.2	2.3 ± 0.2	0.007
GLB		27.4 ± 5.0	29.0 ± 5.3	0.108
HDL-C		1.3 ± 0.4	1.2 ± 0.3	0.535
LDL-C		3.2 ± 1.4	3.4 ± 1.6	0.538
P		1.3 ± 0.4	1.4 ± 0.3	0.664
TC		5.4 ± 1.9	5.7 ± 2.2	0.430
TG		2.0 ± 1.5	2.4 ± 2.0	0.251
TP		67.0 ± 9.3	70.7 ± 9.2	0.036
UA		424.1 ± 112.0	463.2 ± 120.6	0.041
Urea		13.2 ± 9.5	13.5 ± 8.1	0.829
eGFR		44.4 ± 33.0	42.1 ± 29.9	0.628
尿潜血	–	68 (24.3%)	6 (12.5%)	0.070
尿潜血	1+	42 (15.0%)	11 (22.9%)	
尿潜血	2+	41 (14.6%)	5 (10.4%)	
尿潜血	3+	29 (10.4%)	8 (16.7%)	
尿潜血	±	24 (8.6%)	1 (2.1%)	
尿蛋白	–	23 (8.2%)	3 (6.2%)	0.883
尿蛋白	1+	38 (13.6%)	8 (16.7%)	
尿蛋白	2+	73 (26.1%)	8 (16.7%)	
尿蛋白	3+	54 (19.3%)	9 (18.8%)	
尿蛋白	4+	6 (2.1%)	1 (2.1%)	
尿蛋白	±	10 (3.6%)	2 (4.2%)	
居住地	三明	6 (2.1%)	0 (0.0%)	0.970
居住地	南平	20 (7.1%)	3 (6.2%)	
居住地	厦门	7 (2.5%)	1 (2.1%)	
居住地	宁德	13 (4.6%)	1 (2.1%)	
居住地	河南	1 (0.4%)	0 (0.0%)	
居住地	泉州	18 (6.4%)	2 (4.2%)	
居住地	浙江	1 (0.4%)	0 (0.0%)	
居住地	漳州	7 (2.5%)	2 (4.2%)	
居住地	福州	173 (61.8%)	17 (35.4%)	
居住地	莆田	14 (5.0%)	1 (2.1%)	
居住地	贵州	1 (0.4%)	0 (0.0%)	
居住地	龙岩	4 (1.4%)	1 (2.1%)	
年龄		54.4 ± 13.7	55.1 ± 14.8	0.764
性别	女	115 (41.1%)	23 (47.9%)	0.466
性别	男	165 (58.9%)	25 (52.1%)	
糖尿病	–	207 (73.9%)	28 (58.3%)	0.000
糖尿病	无	0 (0.0%)	3 (6.2%)	
糖尿病	有	73 (26.1%)	17 (35.4%)	
肾病类型	IgA肾病	22 (7.9%)	3 (6.2%)	0.218
肾病类型	IgA肾病	0 (0.0%)	1 (2.1%)	
肾病类型	慢性肾炎	40 (14.3%)	10 (20.8%)	
肾病类型	慢性肾衰	212 (75.7%)	34 (70.8%)	
肾病类型	紫癜性肾炎	1 (0.4%)	0 (0.0%)	
肾病类型	紫癜肾炎	1 (0.4%)	0 (0.0%)	
肾病类型	肾病综合征	4 (1.4%)	0 (0.0%)	
高血压	–	167 (59.6%)	19 (39.6%)	0.000
高血压	–	1 (0.4%)	0 (0.0%)	
高血压	无	0 (0.0%)	5 (10.4%)	
高血压	有	112 (40.0%)	24 (50.0%)	

**Table 2:** Comparison of laboratory and clinical variables between CKD G1–2 and CKD G3a–5 in the development cohort.

Variable	Level	G1–2	G3a–5	p-value
A/G		1.5 ± 0.4	1.5 ± 0.3	0.861
ALB		39.5 ± 8.7	39.7 ± 5.6	0.846
ApoA1		1.6 ± 0.4	1.3 ± 0.3	0.000
ApoB		1.3 ± 0.5	1.0 ± 0.4	0.001
CREA		83.2 ± 23.4	311.3 ± 212.6	0.000
Ca		2.2 ± 0.3	2.2 ± 0.2	0.728
GLB		27.0 ± 5.2	27.6 ± 4.8	0.448
HDL-C		1.4 ± 0.5	1.2 ± 0.4	0.049
LDL-C		3.8 ± 1.6	3.0 ± 1.2	0.001
P		1.2 ± 0.2	1.4 ± 0.4	0.000
TC		6.2 ± 2.2	5.0 ± 1.6	0.000
TG		2.3 ± 2.0	1.8 ± 1.1	0.036
TP		66.1 ± 11.7	67.4 ± 7.9	0.392
UA		389.7 ± 102.1	439.2 ± 113.0	0.000
Urea		5.7 ± 1.8	16.4 ± 9.7	0.000
eGFR		87.2 ± 20.5	26.1 ± 16.1	0.000
尿潜血	–	20 (23.8%)	48 (24.5%)	0.033
尿潜血	1+	14 (16.7%)	28 (14.3%)	
尿潜血	2+	16 (19.0%)	25 (12.8%)	
尿潜血	3+	18 (21.4%)	11 (5.6%)	
尿潜血	±	7 (8.3%)	17 (8.7%)	
尿蛋白	–	9 (10.7%)	14 (7.1%)	0.751
尿蛋白	1+	14 (16.7%)	24 (12.2%)	
尿蛋白	2+	25 (29.8%)	48 (24.5%)	
尿蛋白	3+	19 (22.6%)	35 (17.9%)	
尿蛋白	4+	2 (2.4%)	4 (2.0%)	
尿蛋白	±	6 (7.1%)	4 (2.0%)	
居住地	三明	1 (1.2%)	5 (2.6%)	0.187
居住地	南平	2 (2.4%)	18 (9.2%)	
居住地	厦门	3 (3.6%)	4 (2.0%)	
居住地	宁德	5 (6.0%)	8 (4.1%)	
居住地	河南	0 (0.0%)	1 (0.5%)	
居住地	泉州	6 (7.1%)	12 (6.1%)	
居住地	浙江	0 (0.0%)	1 (0.5%)	
居住地	漳州	0 (0.0%)	7 (3.6%)	
居住地	福州	56 (66.7%)	117 (59.7%)	
居住地	莆田	7 (8.3%)	7 (3.6%)	
居住地	贵州	1 (1.2%)	0 (0.0%)	
居住地	龙岩	2 (2.4%)	2 (1.0%)	
年龄		47.1 ± 13.0	57.5 ± 12.8	0.000
性别	女	37 (44.0%)	78 (39.8%)	0.596
性别	男	47 (56.0%)	118 (60.2%)	
糖尿病	–	73 (86.9%)	134 (68.4%)	0.002
糖尿病	有	11 (13.1%)	62 (31.6%)	
肾病类型	IgA肾病	11 (13.1%)	11 (5.6%)	0.000
肾病类型	慢性肾炎	39 (46.4%)	1 (0.5%)	
肾病类型	慢性肾衰	29 (34.5%)	183 (93.4%)	
肾病类型	紫癜性肾炎	0 (0.0%)	1 (0.5%)	
肾病类型	紫癜肾炎	1 (1.2%)	0 (0.0%)	
肾病类型	肾病综合征	4 (4.8%)	0 (0.0%)	
高血压	–	66 (78.6%)	101 (51.5%)	0.000
高血压	–	0 (0.0%)	1 (0.5%)	
高血压	有	18 (21.4%)	94 (48.0%)	

**Figure 1:**
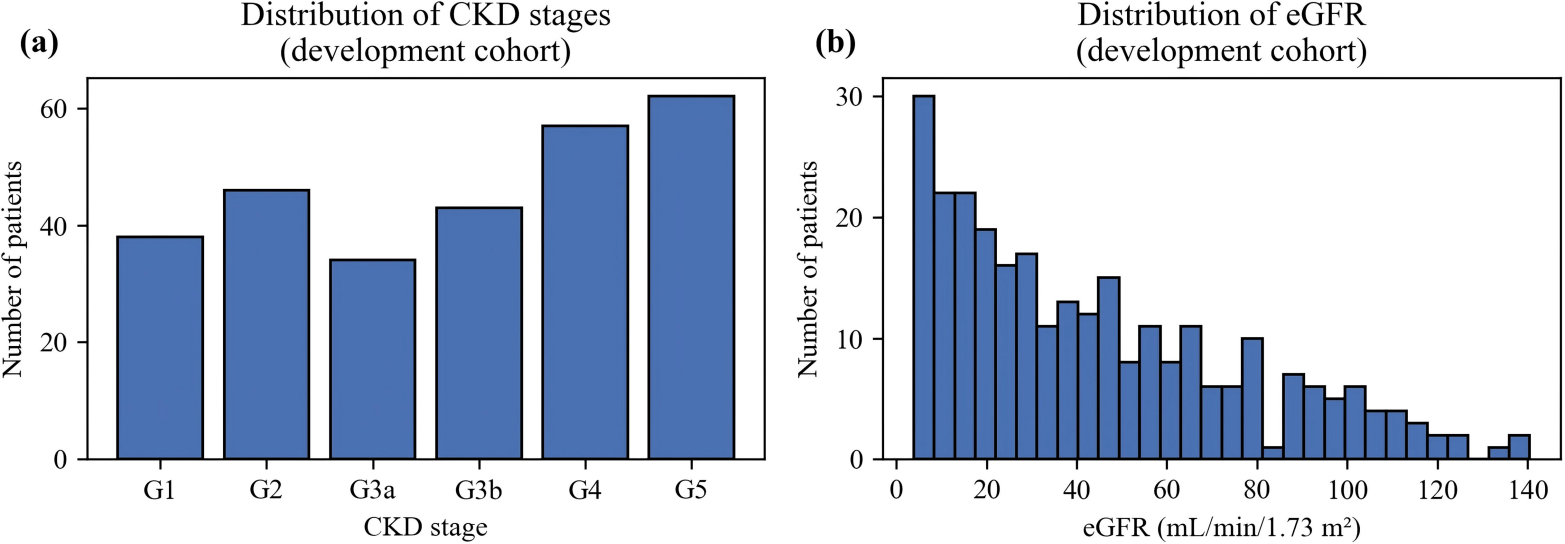
Distribution of CKD stages and eGFR in the development cohort. **(a)** Distribution of CKD stages (G1–G5) in the development cohort. **(b)** Histogram of estimated glomerular filtration rate (eGFR).

The distribution of CKD stages in the development cohort is illustrated in **Figure 1a**. All KDIGO stages (G1-G5) were represented. Patients with advanced CKD (G4-G5) accounted for a relatively greater proportion than those with preserved kidney function (G1-G2), whereas stages G2-G3b contributed intermediate proportions. Consistent with this pattern, the eGFR distribution (**Figure 1b**) exhibited right-skewness, with most patients clustering in the moderate-to-severe impairment range and only a small subset displaying eGFR≥90 mL/min/1.73 m^2^.

**Figure 2:**
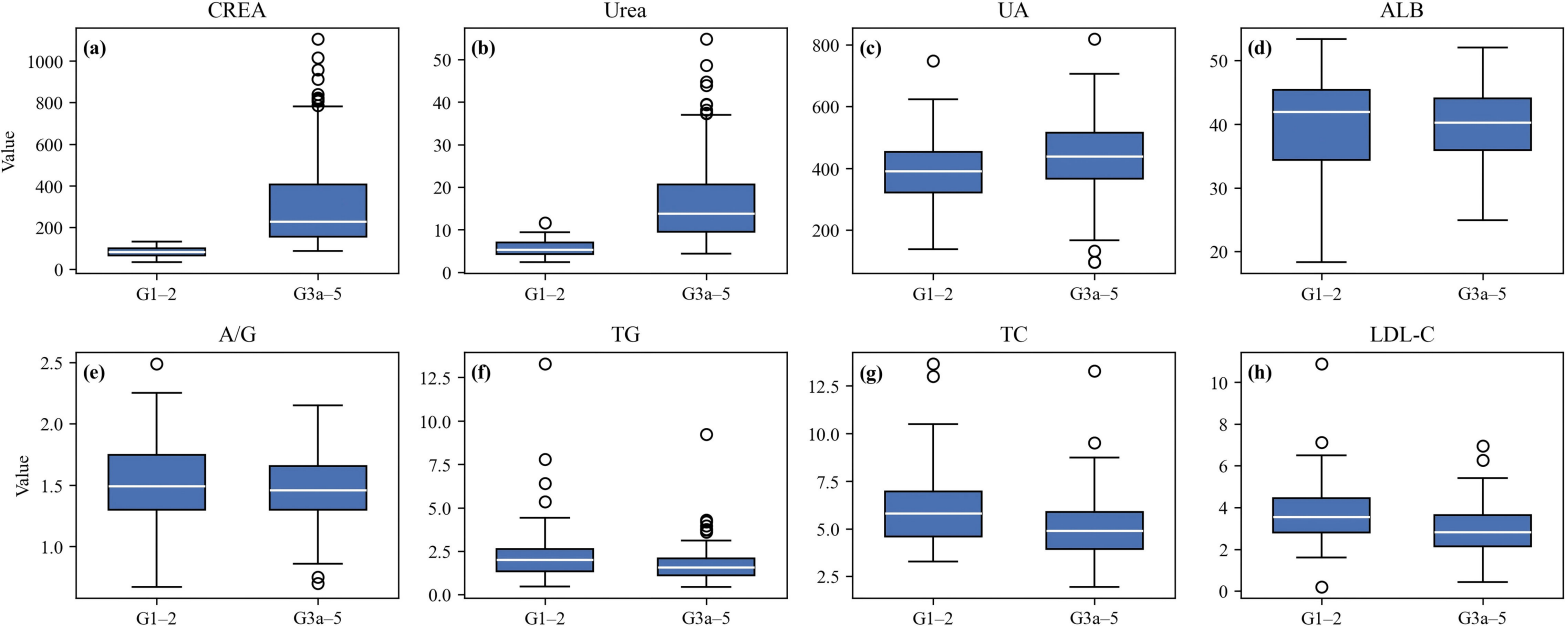
Comparison of laboratory markers between preserved kidney function (G1–2) and advanced CKD (G3a–5). **(a)** CREA, **(b)** Urea, **(c)** UA, **(d)** ALB, **(e)** A/G ratio, **(f)** TG, **(g)** TC, **(h)** LDL-C.

Within the development cohort, patients were further stratified into CKD G1–2 and CKD G3a-5 subgroups. As shown in **Figure 2** and detailed in **Table 2**, markers of kidney function differed markedly between the groups. Serum creatinine, urea, and uric acid were substantially higher in G3a-5 (**Figures 2a–c**), consistent with progressive loss of filtration and metabolic clearance (Delanaye et al., 2020). Conversely, serum albumin and the albumin-to-globulin ratio were lower in G3a-5 (**Figures 2d, e**), reflecting inflammation and nutritional decline documented in advanced CKD. Lipid markers-triglycerides, total cholesterol, and LDL-C-were lower in G3a-G5 (**Figures 2f, h**), aligning with the phenomenon of “reverse epidemiology” in advanced CKD populations.

### 3.2 Diagnostic performance of machine learning models for CKD G3a–5

**Figure 3:**
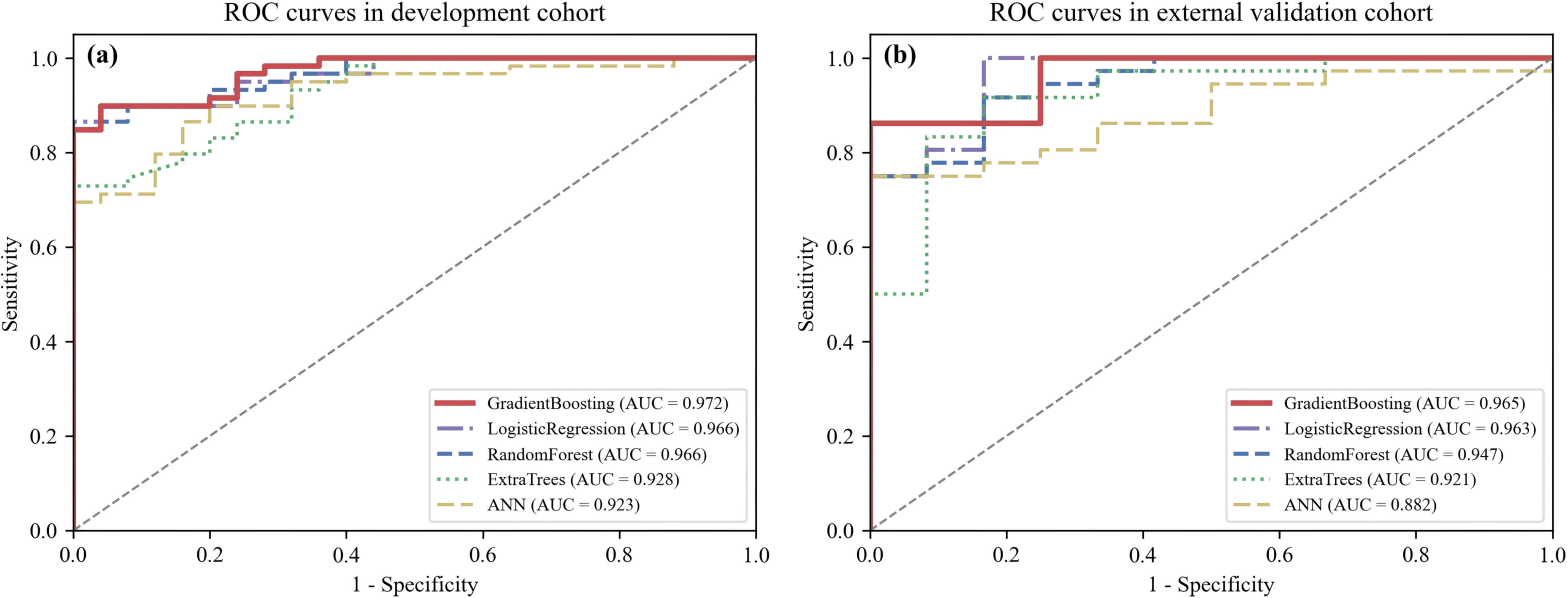
Receiver operating characteristic (ROC) curves of five machine learning models. **(a)** ROC curves in the development cohort. **(b)** ROC curves in the external validation cohort.

The diagnostic performance of the five machine learning algorithms for identifying CKD G3a-5 is summarized in **Table 3** and visualized by ROC curves in **Figure 3**. In the development cohort, all models demonstrated strong discrimination, with AUC values exceeding 0.90 (**Figure 3a**). Gradient Boosting achieved the highest AUC (0.972), closely followed by logistic regression and random forest (AUCs 0.966 for both). Extra Trees and ANN performed slightly less well but still achieved acceptable discrimination, with AUCs of 0.928 and 0.923, respectively.

**Table 3:** Performance metrics of machine learning models in the development cohort and external validation cohort.

Model	AUC_dev	Accuracy_dev	Precision_dev	Recall_dev	F1-score_dev	Kappa_dev	AUC_val	Accuracy_val	Precision_val	Recall_val	F1-score_val	Kappa_val
RandomForest	0.966	0.893	0.946	0.898	0.922	0.752	0.947	0.896	0.919	0.944	0.932	0.714
GradientBoosting	0.972	0.881	0.980	0.847	0.909	0.739	0.965	0.854	0.939	0.861	0.899	0.641
ExtraTrees	0.928	0.857	0.862	0.949	0.903	0.633	0.921	0.875	0.917	0.917	0.917	0.667
LogisticRegression	0.966	0.905	0.964	0.898	0.930	0.782	0.963	0.875	0.941	0.889	0.914	0.684
ANN	0.923	0.845	0.926	0.847	0.885	0.650	0.882	0.771	1.000	0.694	0.820	0.532

External validation results were consistent with the internal findings (**Figure 3b**). Gradient Boosting again produced the highest AUC (0.965), with logistic regression performing similarly (0.963). Random forest (0.947) and Extra Trees (0.921) maintained strong discrimination, whereas ANN exhibited a more pronounced performance drop (AUC 0.882). Performance metrics—including accuracy, precision, recall, F1-score, and Cohen’s kappa—are reported in **Table 3** and followed patterns similar to the AUC results. Gradient Boosting provided the most balanced performance across metrics, while logistic regression and random forest also demonstrated robustness across both cohorts.

**Figure 4:**
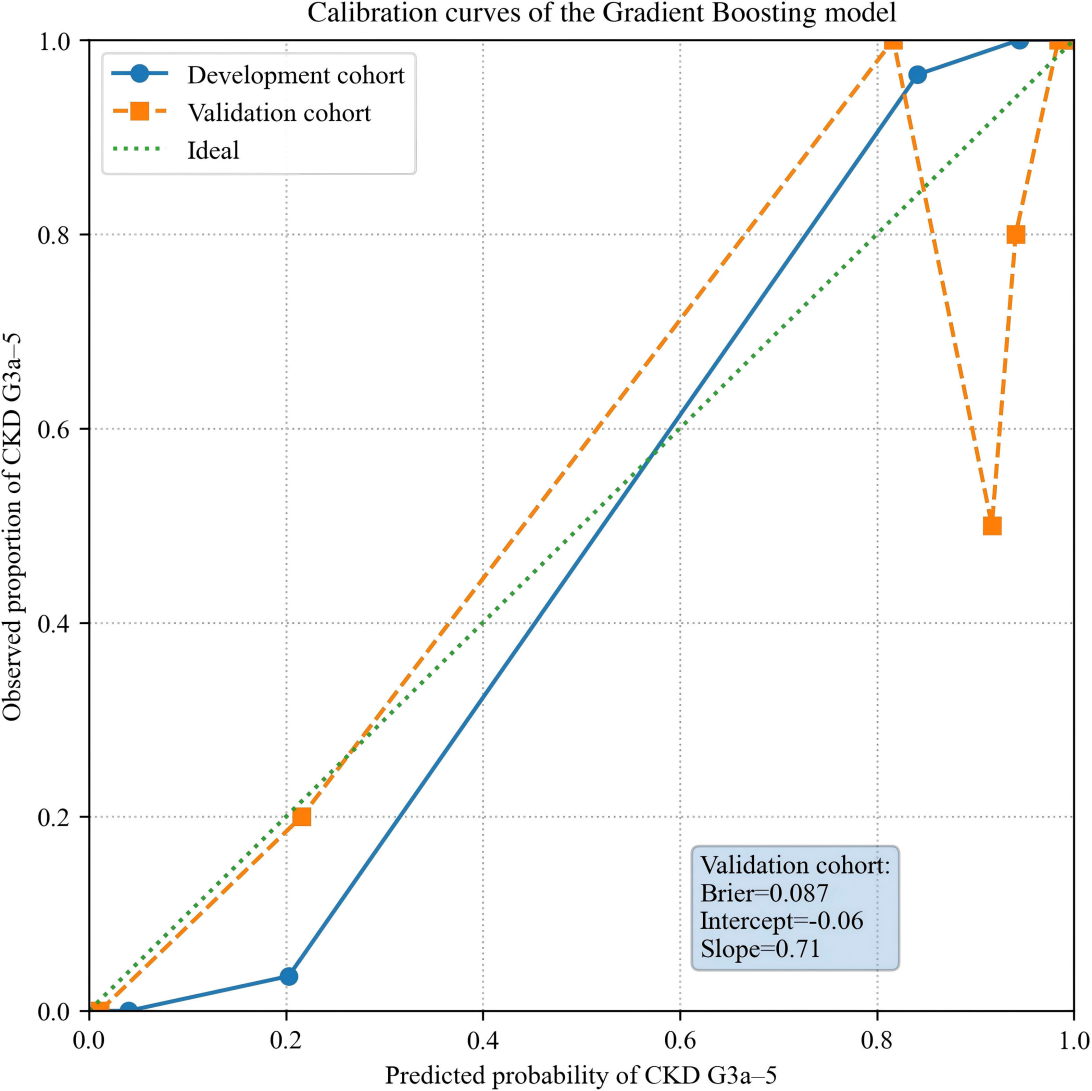
Calibration curves of the Gradient Boosting model.

Calibration of the Gradient Boosting model is shown in **Figure 4**. In the development cohort, the calibration curve closely aligned with the 45°reference line across all risk deciles, indicating strong agreement between predicted and observed probabilities. In the external validation cohort, calibration remained acceptable overall but showed slight underestimation in the mid-range of predicted probabilities. Despite this modest deviation, the Gradient Boosting model maintained adequate calibration for clinical application.

To further explore the impact of decision threshold selection, we conducted a threshold-sensitivity analysis for the Gradient Boosting model in the external validation cohort. As shown in **Table 4**, sensitivity, specificity, PPV, NPV, and Youden index were evaluated across probability thresholds ranging from 0.05 to 0.95. The results indicate that model performance remains relatively stable across a wide range of thresholds. A threshold of 0.5 lies within a plateau region characterized by balanced sensitivity and specificity and a high Youden index, supporting its use as a reasonable reference threshold in this study.

**Table 4:** Performance metrics of the Gradient Boosting model across different probability thresholds in the external validation cohort.

Model	Cohort	Threshold	Sensitivity	Specificity	PPV	NPV	Youden
GradientBoosting	External validation	0.05	0.944	0.75	0.919	0.818	0.694
GradientBoosting	External validation	0.1	0.944	0.75	0.919	0.818	0.694
GradientBoosting	External validation	0.15	0.889	0.75	0.914	0.692	0.639
GradientBoosting	External validation	0.2	0.861	0.75	0.912	0.643	0.611
GradientBoosting	External validation	0.25	0.861	0.833	0.939	0.667	0.694
GradientBoosting	External validation	0.3	0.861	0.833	0.939	0.667	0.694
GradientBoosting	External validation	0.35	0.861	0.833	0.939	0.667	0.694
GradientBoosting	External validation	0.4	0.861	0.833	0.939	0.667	0.694
GradientBoosting	External validation	0.45	0.861	0.833	0.939	0.667	0.694
GradientBoosting	External validation	0.5	0.861	0.833	0.939	0.667	0.694
GradientBoosting	External validation	0.55	0.861	0.833	0.939	0.667	0.694
GradientBoosting	External validation	0.6	0.861	0.833	0.939	0.667	0.694
GradientBoosting	External validation	0.65	0.861	0.833	0.939	0.667	0.694
GradientBoosting	External validation	0.7	0.861	0.833	0.939	0.667	0.694
GradientBoosting	External validation	0.75	0.861	0.833	0.939	0.667	0.694
GradientBoosting	External validation	0.8	0.861	0.833	0.939	0.667	0.694
GradientBoosting	External validation	0.85	0.861	0.833	0.939	0.667	0.694
GradientBoosting	External validation	0.9	0.861	0.833	0.939	0.667	0.694
GradientBoosting	External validation	0.95	0.861	0.833	0.939	0.667	0.694

### 3.3 Feature importance and model interpretation

**Figure 5:**
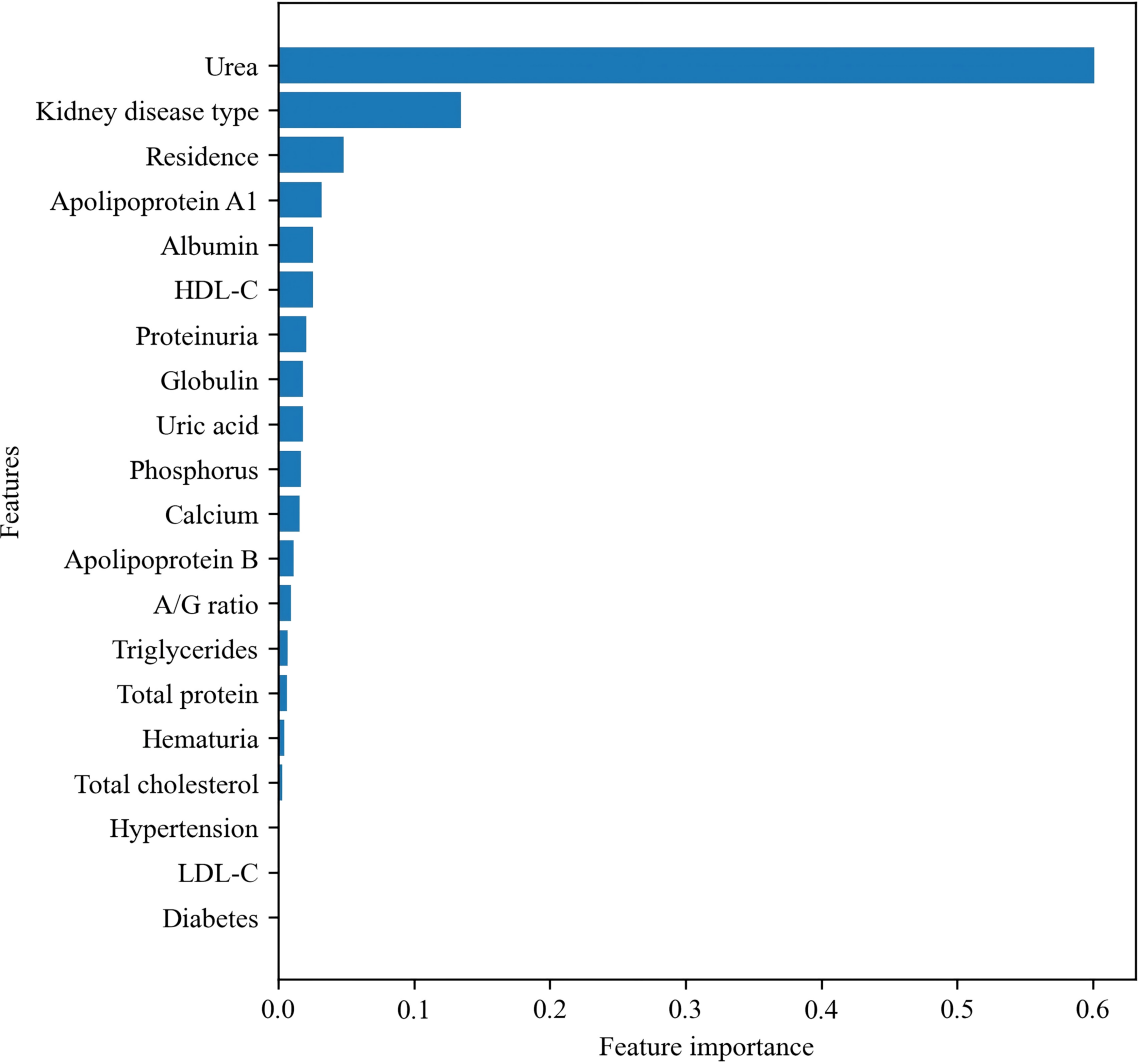
Feature importance ranking of the Gradient Boosting classifier (top 20 features).

To better understand the behavior of the best-performing algorithm, we examined feature importance and SHAP-based model interpretation for the Gradient Boosting classifier. Impurity-based feature importance-after grouping multi-level categorical variables such as kidney disease type and residence-identified urea as the most influential predictor by a substantial margin (**Figure 5**). This finding is consistent with prior work demonstrating urea’s strong association with renal clearance, protein catabolism, and metabolic stress in advanced CKD (48). Kidney disease type ranked second, followed by residence, apolipoprotein A1, albumin, and HDL-C. Additional biochemical features-including uric acid, phosphorus, calcium, globulin, total protein, triglycerides, total cholesterol, and LDL-C-also contributed meaningfully, aligning with established metabolic, inflammatory, and mineral-bone alterations described in CKD progression (49, 50).

**Figure 6:**
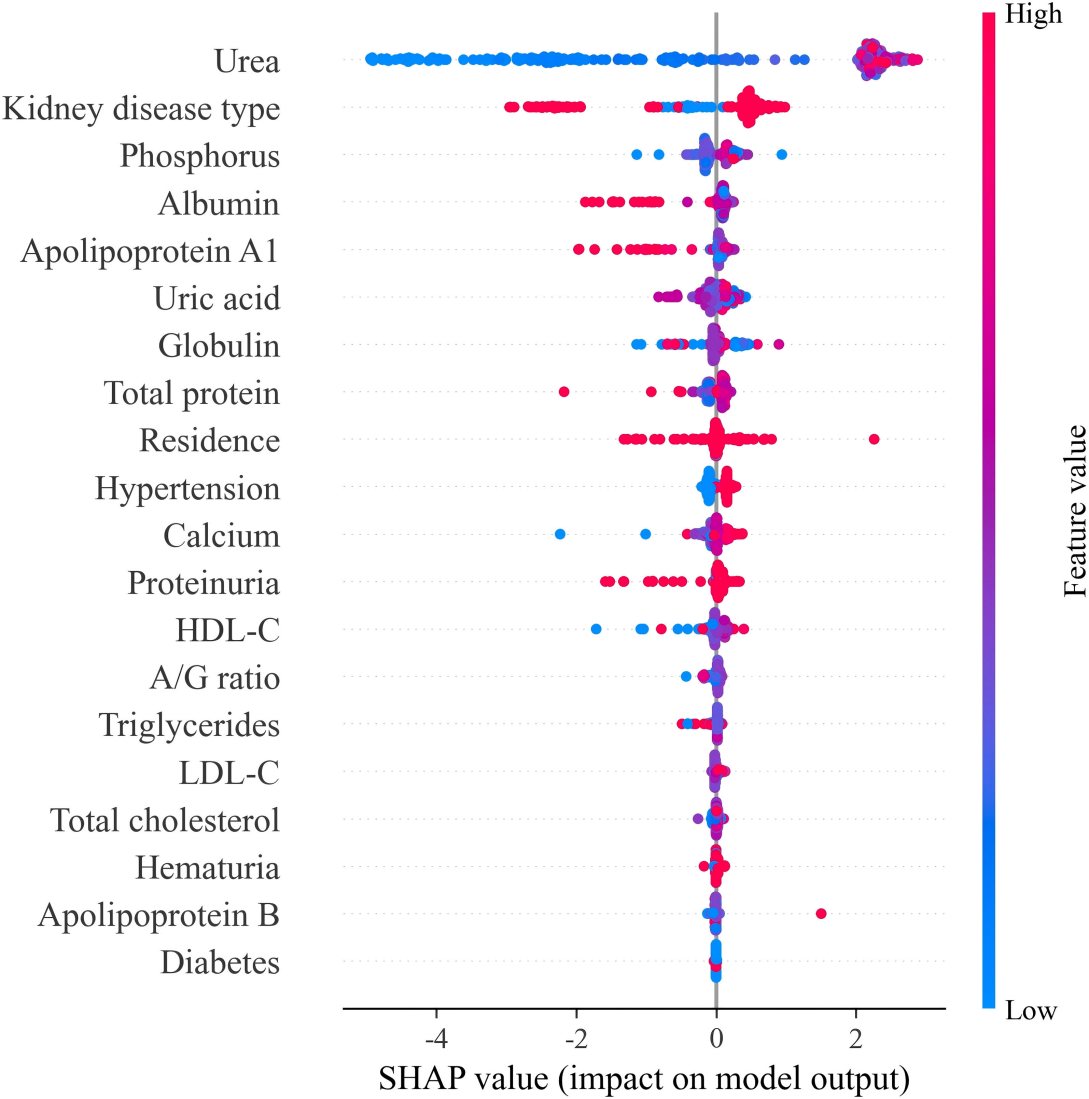
SHAP summary plot showing feature contributions to the Gradient Boosting model.

Global SHAP values (**Figure 6**) revealed clinically coherent patterns. Higher urea consistently produced large positive SHAP contributions, substantially increasing the predicted probability of CKD G3a-G5. Elevated phosphorus and uric acid similarly pushed predictions toward advanced CKD, reflecting CKD-MBD pathophysiology (51) and impaired solute handling. Conversely, higher HDL-C, higher albumin-to-globulin ratio, and-though to a lesser extent-higher total cholesterol and LDL-C tended to reduce predicted risk (52), consistent with the documented “reverse epidemiology” phenomenon in CKD (53, 54).

**Figure 7:**
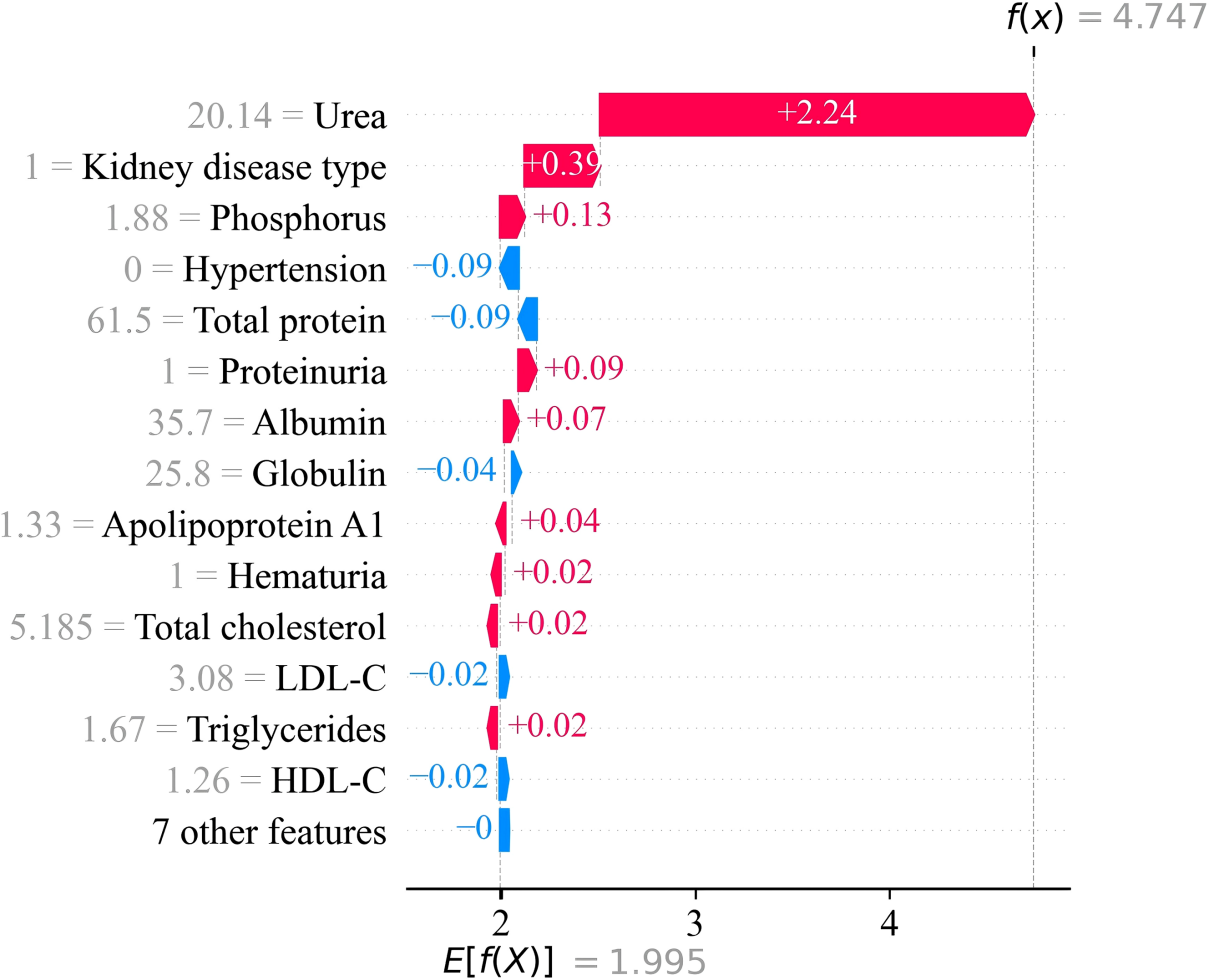
SHAP waterfall plot illustrating feature contributions for a representative patient.

Individual-level interpretability was demonstrated using SHAP waterfall plots (**Figure 7**). In a representative patient, very high urea contributed the largest positive shift in predicted risk, while additional contributors included kidney disease type, phosphorus, and proteinuria. Negative contributions from albumin, total protein, LDL-C, and triglycerides partially offset these effects, but not enough to counteract the dominant influence of urea and renal pathology. These individualized explanations underscore how routine laboratory markers collectively shape risk predictions and confirm that the model’s reasoning aligns with known CKD biology and clinical expectations (46, 55).

From a clinical perspective, such individualized SHAP explanations can be used to support transparent risk communication and to suggest targeted next steps. For example, a prediction driven predominantly by urea and phosphorus may prompt closer evaluation of uremic burden and CKD–mineral bone disorder, whereas a profile driven by low albumin or protein-related indices may highlight the need for nutritional and inflammation assessment. Thus, SHAP helps translate model outputs into patient-specific, clinically interpretable drivers that can be reviewed alongside standard clinical judgment.

### 3.4 Calibration of the Gradient Boosting model

The calibration performance of the Gradient Boosting classifier is presented in **Figure 4**. In the development cohort, the calibration curve closely overlapped with the 45°identity line across the full range of predicted probabilities, indicating excellent agreement between predicted and observed risks of CKD G3a-G5. Such strong calibration enhances the clinical interpretability of the predicted probabilities, in line with recommendations for deploying predictive tools in practice (41).

In the external validation cohort, calibration remained generally acceptable, though the model slightly underestimated risk in the mid-probability range. Modest deviations of this nature are common when applying ML models to independent populations that differ slightly from their development datasets (42, 56). At high-risk levels, predicted and observed rates again aligned closely. Despite these minor discrepancies, overall calibration remained clinically adequate, supporting the Gradient Boosting model’s potential for risk stratification, early detection workflows, and downstream referral prioritization in real-world settings.

Quantitative calibration metrics complemented the visual assessment.In the external validation cohort, the Brier score was 0.028 and the calibration slope was 0.71. A slope below 1.0 indicates moderate shrinkage of predicted risks when applied to an independent dataset, a common finding in external validation studies of moderate-sized cohorts. These quantitative results are consistent with the slight underestimation observed in the mid-probability range in **Figure 4**.

## 4 Discussion

### 4.1 Principal findings and interpretation

In this study, we developed and validated multiple machine learning models to identify advanced CKD (G3a-G5) using routine clinical and laboratory variables. Among all algorithms tested, the Gradient Boosting classifier consistently demonstrated the highest discrimination, achieving AUCs of 0.972 in the development cohort and 0.965 in the external validation cohort (**Figure 3**). Importantly, this strong performance was achieved using routinely measured metabolic laboratory parameters, underscoring the value of capturing systemic endocrine-metabolic dysregulation rather than relying solely on traditional kidney-centric markers. Logistic regression and random forest also performed strongly, whereas the ANN model exhibited reduced generalizability, a limitation commonly observed when neural networks are trained on modestly sized tabular clinical datasets (31).

Interpretability analyses yielded several clinically meaningful insights. Urea emerged as the most influential predictor across all models (**Figure 5**). Although traditionally regarded as a marker of renal clearance, urea also reflects protein catabolism, inflammation, metabolic stress, and endocrine alterations that become increasingly prominent as CKD progresses, consistent with prior evidence in advanced CKD populations (57, 58). SHAP summary plots (**Figure 6**) further confirmed urea as the dominant contributor to higher predicted risk. Additional high-impact predictors-including primary kidney disease type, albumin, phosphorus, uric acid, and apolipoprotein A1-reflect the multidimensional biochemical phenotype associated with CKD progression, consistent with well-described disturbances in mineral metabolism, nutrition-inflammation pathways, and lipid remodeling seen across CKD stages (50, 59, 60).

Collectively, these findings suggest that machine learning models can capture a more holistic biochemical signature of advanced CKD beside traditional kidney function markers. By leveraging nonlinear interactions among metabolic, nutritional, inflammatory, and mineral-bone parameters, ML classifiers provide a level of phenotypic resolution that may be overlooked by conventional linear models—highlighting their potential role in augmenting clinical risk assessment and early identification efforts (46, 55).

### 4.2 Comparison with previous literature

Our study builds on and extends prior work applying machine learning approaches to nephrology. Previous studies have demonstrated that tree-based ensemble methods outperform traditional regression models in predicting CKD onset, rapid eGFR decline, or kidney failure due to their ability to accommodate nonlinear relationships and complex interactions (31, 61). Our results reinforce this observation: Gradient Boosting and random forest outperformed logistic regression and ANN, particularly in external validation, highlighting their robustness for clinical prediction using heterogeneous laboratory data.

The identified key predictors also align with established CKD biology. Urea has been increasingly recognized as a marker of both reduced renal clearance and heightened protein catabolism-components of the “malnutrition-inflammation-atherosclerosis (MIA) syndrome” prevalent in advanced CKD (57, 62). Albumin, a sensitive marker of nutritional status and chronic inflammation, has well-established associations with CKD progression and mortality (50). Elevated phosphorus reflects early disturbances in mineral metabolism and is a hallmark of CKD-mineral bone disorder (CKD-MBD). Lipid-related markers (HDL-C, apolipoprotein A1) showed protective SHAP effects, which is consistent with the paradoxical “reverse epidemiology” observed in CKD, where lower cholesterol levels are often associated with worse outcomes due to malnutrition and systemic inflammation (63). These findings indicate that lipid- and protein-related parameters in advanced CKD should be interpreted within an endocrine-metabolic and nutrition-inflammation framework, rather than through conventional cardiovascular risk paradigms.

Additionally, primary kidney disease type had substantial predictive value. This reflects the heterogeneity of CKD pathophysiology (64), as glomerular diseases, diabetic kidney disease, and hypertensive nephrosclerosis display distinct trajectories of nephron loss and biochemical alteration. By capturing these subtle differences, machine learning models further improve upon the limitations of risk stratification based solely on eGFR.

Importantly, our study includes an external validation cohort, which many prior studies lack. The preservation of model performance across cohorts provides supportive evidence for the generalizability of the Gradient Boosting classifier. However, given the relatively modest size of the external validation cohort, these findings should be interpreted with caution and warrant confirmation in larger, multicenter populations.

### 4.3 Clinical implications

Additionally, the substantial predictive value of primary kidney disease type reflects the well-known heterogeneity of CKD pathophysiology. Glomerular diseases, diabetic kidney disease, and hypertensive nephrosclerosis progress along distinct trajectories of nephron loss, inflammatory activation, metabolic alteration, and fibrosis (5, 65). By capturing these nuanced phenotypic signatures, machine learning models provide a more refined risk stratification framework than eGFR-based staging alone.

Importantly, the proposed model should be viewed as complementary to, rather than competitive with, conventional eGFR-based staging. While eGFR reflects renal filtration capacity, the machine learning framework captures a broader metabolic phenotype associated with advanced CKD. This complementary information may support earlier identification, referral prioritization, and holistic clinical assessment, particularly in settings where reliance on creatinine-based staging alone may underestimate systemic disease burden.

In practice, such a model could function as a triage and risk stratification tool within specialist nephrology clinics for adult patients with established CKD, using routinely available metabolic panels to support clinical decision-making. It may help identify patients with advanced CKD phenotypes who could benefit from intensified monitoring, CKD-MBD evaluation, or nutritional assessment, particularly in cases where reliance on serum creatinine or eGFR alone may not fully capture the systemic metabolic burden of disease. Unlike eGFR-based staging, which primarily reflects renal filtration capacity, this approach integrates metabolic, mineral, and nutritional signals to characterize a broader metabolic phenotype associated with advanced CKD. Rather than replacing conventional CKD staging, the model is intended to complement existing frameworks by providing additional contextual information within specialist care settings.

In addition, SHAP-based interpretability can facilitate implementation in real-world workflows by linking the predicted risk to actionable biochemical domains. At the population level, global SHAP patterns can help clinicians recognize common metabolic phenotypes of advanced CKD (e.g., azotemia, mineral imbalance, and malnutrition–inflammation signatures), supporting protocol development and quality improvement. At the individual level, patient-specific SHAP explanations can be displayed within the electronic health record to justify why a patient is flagged as high risk and to guide follow-up actions such as repeat kidney function testing, assessment of CKD–MBD parameters, nutritional evaluation, medication review, or referral prioritization. Importantly, these explanations are intended to enhance transparency and clinical communication rather than to infer causality.

### 4.4 Strengths and limitations

This study offers several notable strengths. It incorporates a diverse array of routinely obtained laboratory parameters and clinical variables, enabling multidimensional assessment of CKD severity. The model development process adhered to best practices, including rigorous preprocessing, stratified internal testing, and independent external validation. The inclusion of explainable AI methods provides transparency, addressing one of the major barriers to implementing machine learning in clinical nephrology.

However, several limitations warrant consideration. First, the overall sample size—particularly that of the external validation cohort—was modest. This may have limited the ability to capture rare clinical patterns and contributed to variability in the performance of more complex models, such as the artificial neural network. Accordingly, estimates of external validation performance may be subject to greater uncertainty, and conclusions regarding model generalizability should be regarded as preliminary until confirmed in larger and more heterogeneous populations.In this context, the external calibration slope of 0.71 suggests moderate shrinkage of predicted risks when applied to the independent cohort. A slope below 1.0 reflects some degree of overfitting during model development and is commonly observed in external validation studies with moderate sample sizes and multiple predictors. Importantly, this finding indicates compression of extreme predicted probabilities rather than severe miscalibration, and overall discrimination remained strong in the validation cohort. Furthermore, internal validation was based on a single stratified 70/30 split. Although this approach preserves independence between training and testing subsets, repeated resampling strategies such as k-fold cross-validation or bootstrapping may provide more stable internal performance estimates in moderate-sized datasets. Future studies should incorporate repeated internal validation procedures to further assess model robustness.

In addition, the ratio of sample size to predictor dimensionality—particularly after one-hot encoding of categorical variables—may introduce instability in flexible machine learning models. Although the overall number of effective predictors remained moderate and the dominant predictive signal was driven by continuous laboratory variables, performance estimates and feature importance rankings should be interpreted with caution. Larger multicenter datasets and repeated resampling strategies (e.g., cross-validation or bootstrapping) will be required to further assess model stability and robustness. Moreover, although the external validation cohort was drawn from a different clinical center, partial overlap in the underlying healthcare network—such as involvement of similar specialty teams—may have reduced the degree of complete independence between the development and validation cohorts, underscoring the need for broader multicenter validation.

Second, all data were derived from two centers within a single healthcare system. As a result, the findings may not fully generalize to populations with distinct demographic characteristics, disease spectra, or healthcare delivery structures. Third, missing data were handled using median imputation for continuous variables and an explicit “Missing” category for categorical variables. While this pragmatic strategy preserves sample size and enhances reproducibility in moderate-sized datasets, we did not perform a formal sensitivity analysis comparing alternative imputation methods, which represents an additional limitation. Future studies with larger multicenter cohorts should evaluate whether more advanced imputation approaches materially influence model discrimination, calibration, or interpretability.

Fourth, although SHapley Additive exPlanations (SHAP) improve model transparency, the relationships identified remain associative rather than causal and should not be interpreted as evidence of mechanistic pathways. Finally, the present study focused on cross-sectional classification of advanced CKD; longitudinal modeling will be required to determine whether similar approaches can reliably predict disease progression over time. In addition, certain categorical predictors—such as primary kidney disease type and place of residence—may be system-specific and not uniformly available across healthcare settings, which could affect direct portability of the model.

Finally, the present study focused on cross-sectional classification of advanced CKD at a single time point and was not designed as a prognostic model. Longitudinal prediction of CKD progression will require repeated follow-up measurements and time-dependent outcomes (e.g., sustained eGFR decline, transition to a higher CKD stage, kidney failure, or dialysis initiation), which were not available in the current dataset.

### 4.5 Future directions

Future research should expand the dataset to include larger and more diverse populations, enabling further refinement and validation of the model across different clinical settings. Incorporating additional variables-such as cystatin C, inflammatory biomarkers, medication use, and patient-reported outcomes-may further enhance predictive performance. Extending the approach to longitudinal prediction could provide clinicians with tools for identifying patients at risk of rapid progression or adverse renal outcomes. With longitudinal cohorts, the same routinely measured metabolic laboratory indices could be incorporated as time-varying covariates in survival or trajectory frameworks, such as Cox proportional hazards models, random survival forests, or sequence-based models that leverage repeated laboratory trajectories. This would enable estimation of progression risk over clinically meaningful horizons and support prognostic stratification beyond cross-sectional staging.

Moreover, integrating machine learning algorithms with electronic health systems may enable continuous, automated monitoring of renal function trajectories. Combining structured laboratory data with imaging, pathology, or genomic information may also pave the way toward precision nephrology models capable of individualized treatment planning and targeted intervention strategies.

In conclusion, we developed and externally validated an interpretable machine learning model that integrates routinely available metabolic laboratory data to accurately identify advanced CKD. By capturing clinically meaningful endocrine–metabolic patterns beyond traditional kidney-centric assessment, this approach may facilitate more refined identification, referral prioritization, and risk stratification among patients with established CKD within specialist care settings.

## 5 Conclusions

In this study, we developed and externally validated machine learning models capable of accurately identifying advanced CKD (G3a-G5) using routinely available clinical and biochemical variables. Among all algorithms evaluated, the Gradient Boosting classifier demonstrated the most robust and generalizable performance, supported by excellent discrimination, clinically meaningful calibration, and high interpretability.

Model explainability analyses revealed that key predictors-including urea, primary kidney disease type, markers of mineral metabolism, nutritional status, and lipid-associated biomarkers—collectively capture a multidimensional biochemical signature of advanced renal dysfunction. These findings highlight that machine learning methods can leverage nonlinear interactions across metabolic, inflammatory, and kidney-specific pathways to improve identification of patients with more advanced CKD overcame the limitations of traditional indicators.

The proposed model offers a practical and explainable tool that could be integrated into electronic health systems to support earlier detection of advanced CKD, facilitate timely referral to nephrology, and enhance individualized risk communication. Future work incorporating larger and more diverse populations, additional biomarkers, and longitudinal trajectories will be essential to optimizing model performance and extending its application to CKD progression prediction.

## Data Availability

The original contributions presented in the study are included in the article/supplementary material. Further inquiries can be directed to the corresponding author.
